# Functional connectivity-based signatures of schizophrenia revealed by multiclass pattern analysis of resting-state fMRI from schizophrenic patients and their healthy siblings

**DOI:** 10.1186/1475-925X-12-10

**Published:** 2013-02-07

**Authors:** Yang Yu, Hui Shen, Huiran Zhang, Ling-Li Zeng, Zhimin Xue, Dewen Hu

**Affiliations:** 1 College of Mechatronics and Automation, National University of Defense Technology, Changsha, China; 2 Mental Health Institute of the Second Xiangya Hospital, Hunan province, Technology Institute of Psychiatry, Key Laboratory of Psychiatry and Mental Health of Hunan Province, Central South University, Changsha, China

**Keywords:** Schizophrenia, Healthy siblings, Functional magnetic resonance imaging, Resting-state, Functional connectivity, Multiclass pattern analysis

## Abstract

**Background:**

Recently, a growing number of neuroimaging studies have begun to investigate the brains of schizophrenic patients and their healthy siblings to identify heritable biomarkers of this complex disorder. The objective of this study was to use multiclass pattern analysis to investigate the inheritable characters of schizophrenia at the individual level, by comparing whole-brain resting-state functional connectivity of patients with schizophrenia to their healthy siblings.

**Methods:**

Twenty-four schizophrenic patients, twenty-five healthy siblings and twenty-two matched healthy controls underwent the resting-state functional Magnetic Resonance Imaging (rs-fMRI) scanning. A linear support vector machine along with principal component analysis was used to solve the multi-classification problem. By reconstructing the functional connectivities with high discriminative power, three types of functional connectivity-based signatures were identified: (i) state connectivity patterns, which characterize the nature of disruption in the brain network of patients with schizophrenia; (ii) trait connectivity patterns, reflecting shared connectivities of dysfunction in patients with schizophrenia and their healthy siblings, thereby providing a possible neuroendophenotype and revealing the genetic vulnerability to develop schizophrenia; and (iii) compensatory connectivity patterns, which underlie special brain connectivities by which healthy siblings might compensate for an increased genetic risk for developing schizophrenia.

**Results:**

Our multiclass pattern analysis achieved 62.0% accuracy via leave-one-out cross-validation (p < 0.001). The identified state patterns related to the default mode network, the executive control network and the cerebellum. For the trait patterns, functional connectivities between the cerebellum and the prefrontal lobe, the middle temporal gyrus, the thalamus and the middle temporal poles were identified. Connectivities among the right precuneus, the left middle temporal gyrus, the left angular and the left rectus, as well as connectivities between the cingulate cortex and the left rectus showed higher discriminative power in the compensatory patterns.

**Conclusions:**

Based on our experimental results, we saw some indication of differences in functional connectivity patterns in the healthy siblings of schizophrenic patients compared to other healthy individuals who have no relations with the patients. Our preliminary investigation suggested that the use of resting-state functional connectivities as classification features to discriminate among schizophrenic patients, their healthy siblings and healthy controls is meaningful.

## Background

Schizophrenia is a highly heritable psychiatric disorder, and studies demonstrate that heritable factors may play an important role in the pathogenesis of schizophrenia [1,2]. Indeed, it has been suggested that genetic factors contribute to 80% of the risk of developing schizophrenia [3]. In addition, it is supported that the similar genetic backgrounds between patients with schizophrenia and their healthy siblings result in an approximately nine-fold higher risk for those siblings to develop schizophrenia than that of the general population [4-6]. Thus, comparison among patients with schizophrenia, their healthy siblings and healthy controls should provide acbldditional insight into the pathophysiological mechanism underlying schizophrenia and might be helpful in further highlighting the genetic contribution to the etiology of schizophrenia.

Recently, a growing number of neuroimaging studies have begun to investigate the brains of patients with schizophrenia and their healthy siblings for identifying neuroimaging-based biomarkers of this complex disorder [1,7,8]. Early investigations using voxel-based methods suggested structural and functional abnormalities in some brain regions of patients with schizophrenia [9,10]. Other studies suggested that patients with schizophrenia might suffer from dysfunctional integration between some special brain regions [2,11-16]. Furthermore, evidence from Diffusion Tensor Imaging (DTI) also revealed anatomical disconnection in schizophrenic patients [17]. A recent study using both fMRI and DTI analysis revealed the concurrence of the resting-state functional disconnectivity and damaged anatomical connectivity in schizophrenia [18]. Further findings suggested that resting-state functional connectivity disturbances vary by network in schizophrenia [19,20], whereas, the pathophysiological mechanism of schizophrenia is still uncertain. A recent study suggested that patients with schizophrenia and their healthy siblings both show resting-state network alterations [21]; however, this effect is more difficult to diagnose due to the use of group-level statistical methods. In the current study, we investigated the whole-brain functional connectivity patterns of patients with schizophrenia, their healthy siblings and healthy controls at the individual subject level.

Multivariate pattern analysis has recently aroused great interest for its capacity of finding valuable neuroimaging-based biomarkers [7,11,22]. This analysis also very helpful for clinical diagnosis at the individual level, which can complete previous group-level statistical analysis studies [23]. Machine learning is an important aspect of multivariate pattern analysis, and linear and nonlinear learning algorithms have both been used in pattern analysis of fMRI data [11,24]. Compared with nonlinear algorithms, linear algorithms were argued to be more insensitive to overfitting problems, especially for high feature dimension and small sample size [25]. Another advantage of linear algorithms is that they can reveal potential neuroimaging-based biomarkers using reconstruction technique.

In our previous study, multiclass pattern analysis method was used to investigate the risk for healthy siblings of patients with schizophrenia to develop the disorder based on whole-brain resting-state functional connectivity [4]. However, nonlinear learning algorithm cannot reveal connectivities with high discriminative power which could potentially be neuroimaging-based biomarkers underlying the pathophysiological mechanisms of schizophrenia. In the present study, we used a multiclass linear classifier to explore the whole-brain functional connectivity patterns of patients with schizophrenia, their healthy siblings and healthy controls. Similar to a previous study [26], we defined three types of functional connectivity-based signatures: (i) state connectivity patterns, underlying the nature of the abnormality in the brain network of patients with schizophrenia; (ii) trait connectivity patterns, corresponding to the abnormal functional connectivity shared by patients with schizophrenia and their healthy siblings, providing a possible neuroendophenotype to help bridge genomic complexity and disorder heterogeneity; and (iii) compensatory connectivity patterns, revealing special brain connectivities by which healthy siblings might compensate for an increased genetic risk for developing schizophrenia.

## Methods

### Participants

Subjects consisted of twenty-five patients with schizophrenia, twenty-five healthy siblings and twenty-five healthy controls. Patients with schizophrenia were recruited from outpatient departments and inpatient units at the Second Xiangya Hospital of Central South University. All of the patients fulfilled the criteria for schizophrenia according to the DSM-IV (Diagnostic and Statistical Manual of Mental Disorders, Fourth Edition). Symptom severity for the patients was assessed using the positive and negative syndrome scale [27]. No patients had a history of neurological disorders, substance abuse, or electroconvulsive therapy. Six of the patients with schizophrenia were medication-free, while the others accepted atypical psychotropic drugs during the time of scanning (risperidone [n = 10, 2–6 mg/day], clozapine [n = 4, 200–350 mg/day], quetiapine [n = 4, 400–600 mg/day], and sulpiride [n = 1, 200 mg/day]). Twenty-five healthy siblings who do not fulfill the DSM-IV criteria for any Axis-I psychiatric disorders were recruited so that each schizophrenic patient had a corresponding sibling. The patient with schizophrenia and the corresponding sibling were from the same family (they are sisters or brothers). Twenty-five healthy controls who have no relations with the schizophrenic patients were recruited from Changsha City, China.

One schizophrenic patient and three of the healthy controls were excluded for excessive head motion during scanning acquisition (>1.0 mm translation and/or >2° rotation). The remaining subjects with schizophrenia, healthy siblings, and the healthy controls were demographically similar with respect to age, gender, and education levels (Table 1). All of the participants gave their written informed consent to participate in the study and were studied under protocols approved by the Second Xiangya Hospital of Central South University.

**Table 1:** Demographic and clinical profiles of the participants in this study (Mean ± SD)

**Characteristics**	**Schizophrenia patient(n = 24)**	**Healthy sibling(n = 25)**	**Healthy control(n = 22)**
Sex(males/females)	12/12	15/10	12/11
Education (years)	12.28 ±2.5	12.48 ± 2.52	13.52 ± 2.85
Age (years)	25.36 ±6.32	25.56 ± 6.78	25.48 ± 5.45
PANSS total	87.14 ±12.2		
PANSS positive	21.81 ±4.6		
PANSS negative	22.38 ±5.3		
PANSS general	41.96 ±6.4		

PANSS, Positive and Negative Syndrome Scale.

### Resting experiment and data acquisition

fMRI scans were performed with a 1.5 T GE Signa System (GE Signa, Milwaukee, Wisconsin, USA) via using a gradient-echo echo planar imaging sequence. The imaging parameters are as follows: TR = 2000 ms, TE = 40 ms, FOV = 24 cm, FA = 90°, matrix = 64 × 64, slice thickness = 5 mm, gap = 1 mm, slices = 20. In the experiment, the subjects were instructed to be relaxed, simply to keep their eyes closed and to remain awake and perform no specific cognitive exercise. Foam pads and earplugs were used to minimize head motion and scanner noise, respectively. Each functional resting-state session lasted 6 minutes, resulting in 180 volumes.

### Data preprocessing

Image preprocessing was performed using the statistical parametric mapping software package (SPM8, Welcome Department of Cognitive Neurology, Institute of Neurology, London, UK, http://www.fil.ion.ucl.ac.uk/spm). For each subject, the first 5 volumes of the scanned data were discarded for magnetic saturation effects. The remaining volumes were corrected by registering and reslicing for head movement. Then the volumes were normalized to the standard echo planar imaging template in the Montreal Neurological Institute space. The resulting images were spatially smoothed with a Gaussian filter of 8 mm full-width Fhalf-maximum kernel and then temporally filtered with a Chebyshev band-pass filter (0.01-0.08 Hz). The registered fMRI volumes were further divided into 116 regions according to the anatomically labeled template previously validated and reported by Tzourio-Mazoyer *et al.* (2002).

Regional mean time series were acquired by averaging the fMRI time series over all voxels within each of the 116 regions. We further regressed out the global mean signals and the effects of the head motions. Then we calculated the Pearson’s correlation coefficients between each pair of regions, resulting in a 6670 dimensional feature vector for each subject.

Regional mean time series were acquired for each individual by averaging the fMRI time series over all voxels within each of the 116 regions. For each regional mean time series, we further regressed out the global mean signals and the effects of translations and rotations of the head estimated in the course of initial movement correction by image realignment. The residuals of the above regressions constituted the set of regional mean time series used for further functional connectivity analysis [28]. Then we calculated the Pearson’s correlation coefficients between each pair of regions, resulting in a 6670 dimensional feature vector representing the resting-state functional network for each subject.

### Multiclass pattern analysis

**Figure 1:**
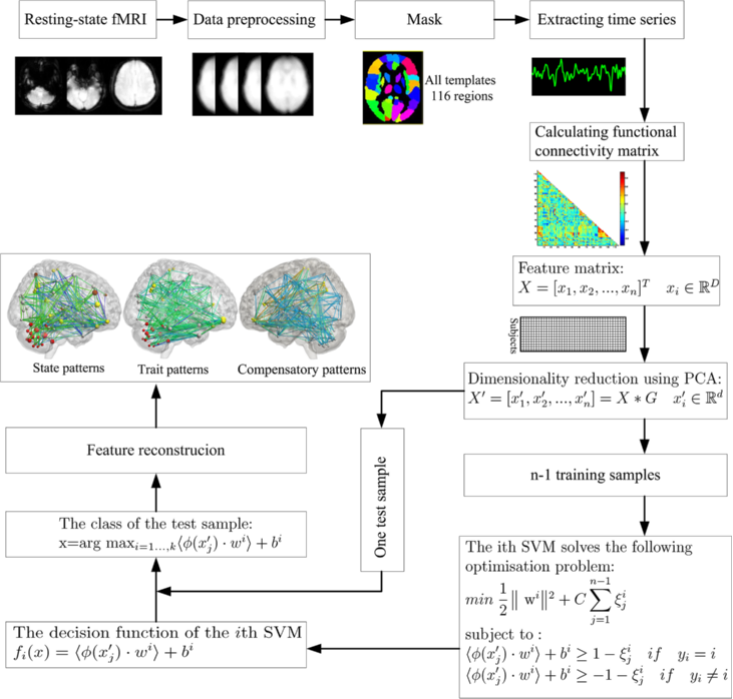
Flowchart of our method.

Principal component analysis (PCA) was applied to reduce the dimensionality of original feature space [29]. When the dataset of features with high discriminative power was obtained, support vector machines (SVMs) with linear kernel function were employed to solve the classification problem [30]. The one-against-rest strategy was used in designing our classifiers [31]. For a *k*-class problem, the one-against-rest method constructed *k* SVM models (Figure 1). The *i*th SVM is trained with the training samples in the *i*th class with positive labels and other samples with negative labels. The final output of the one-against-rest method is the class that corresponds to the SVM with the highest output value. The leave-one-out cross-validation (LOOCV) strategy was employed to estimate the generalization ability of our classifiers [11]. Statistical significance of the classification accuracy was determined by permutation tests [23,32]. In permutation testing, the class labels of the training data were randomly permuted prior to training. Cross-validation was then performed on the permuted training set, and the permutation was repeated 1,000 times. It was assumed that a classifier learned reliably from the data when the generalization rate obtained by the classifier trained on the real class labels exceeded the 95% confidence interval of the classifier trained on randomly relabeled class labels.

### Identification of features with high discriminative power

We determined the functional connectivity features with the highest discriminative power by reconstruction based on the performance of each one-against-rest classifier. Because each feature influences the classification via its weight, the larger the absolute magnitude of a feature’s weight is, the stronger it will affect the classification result. For each one-to-rest classifier, we obtained a weight vector in each LOOCV experiment. The weight vector for the one-against-rest classifier was finally acquired by averaging these above weight vectors. We therefore obtained three weight vectors representing the features’ discriminative power for each one-against-rest classifier. Because we performed the classification in the dimension-reduced subspace, to determine the original functional connectivities which make significantly contributions to the classification, we used the method detailed in a previous study [33] to map back each weight vector to the original high-dimensional space. Thus, for all of the 6670 resting-state functional connectivities, we obtained the order of their contribution to the classification for each one-against-rest classifier.

## Results

### Classification results

Multiclass pattern analysis was employed to perform classification among these three groups, resulting in an accuracy of 62.0% by LOOCV. The correct classifications of the patients with schizophrenia, the healthy siblings, and the healthy controls were 66.7%, 56%, 63.6%, respectively (Table 2). The permutation test results indicated that the classifier learned the relationship between the data and the labels with a probability of error of <0.001.

**Table 2:** Confusion matrix for results in leave-one-out cross-validation

**Classes**	**Schizophrenia**	**Healthy siblings**	**Healthy controls**
Schizophrenia	66.7%	12.5%	20.8%
Healthy siblings	16.0%	56.0%	28.0%
Healthy controls	9.1%	27.3%	63.6%

The rows of this matrix indicate the groups of the subjects (ground truth), and the columns indicate the predictions by the classifier. The cells in each row contain the proportion of trials in which subjects responded with the category indicated by the column.

### Functional connectivity with high discriminative power

**Figure 2:**
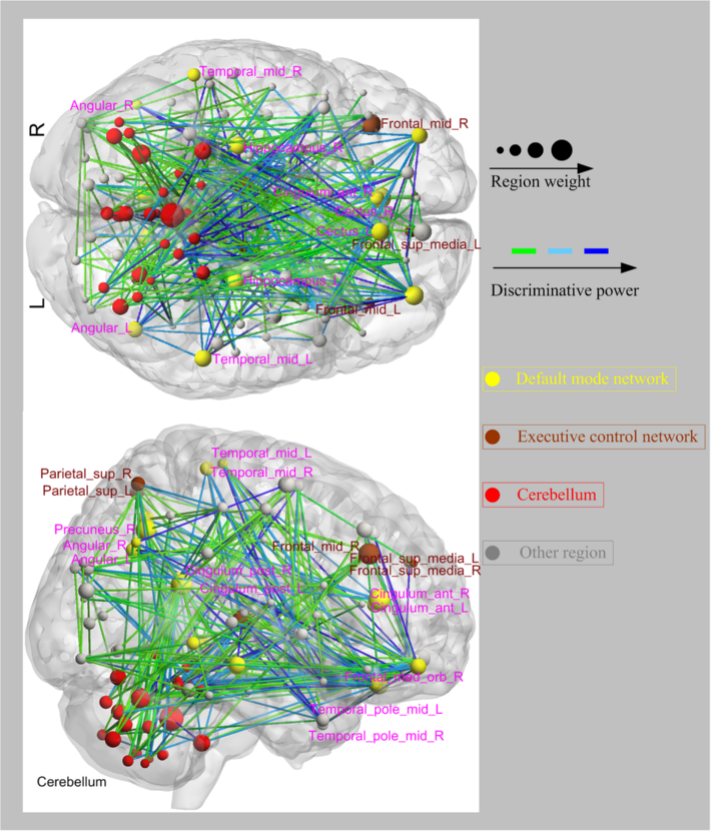
**Regions weights and the distribution of the 330 discriminative functional connectivities responding to the state patterns in bottom and right view.** Regions are color-coded by category. The line colors represent the discriminative power of the connectivities.

**Figure 3:**
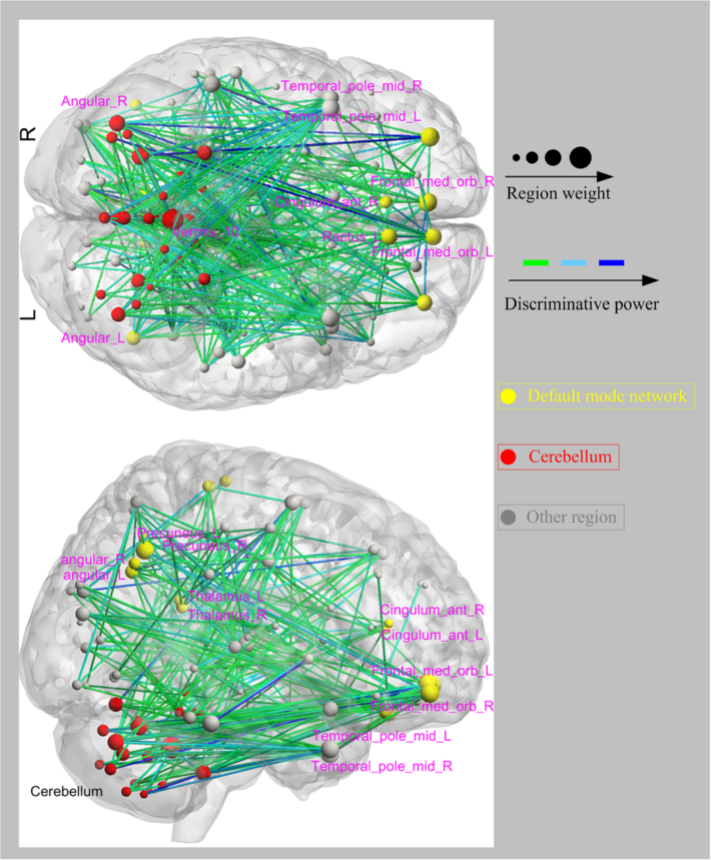
**Regions weights and the distribution of the 330 discriminative functional connectivities responding to the trait patterns in bottom and right view.** Regions are color-coded by category. The line colors represent the discriminative power of the connectivities.

**Figure 4:**
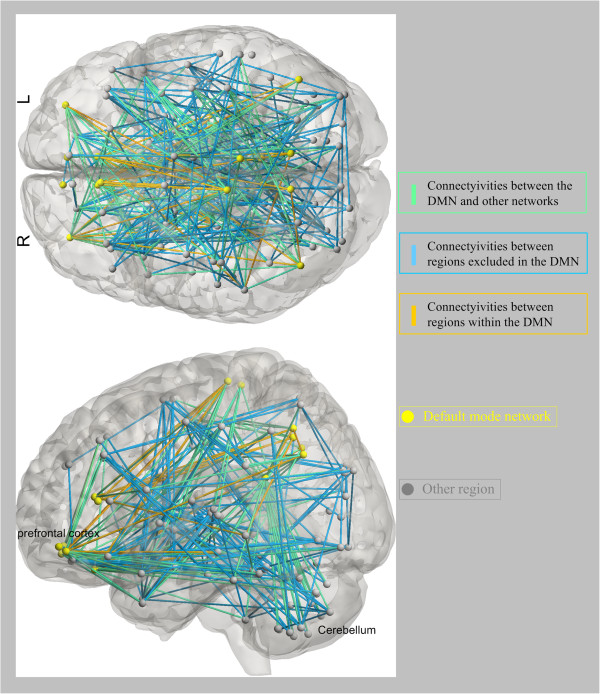
**Regions and the distribution of the 330 discriminative functional connectivities responding to the compensatory patterns in top and left view.** Regions are color-coded by category. The lines are color-coded by the regions the connectivities associated with.

We selected 5% of total functional connectivities with the highest discriminative power from each one-against-rest classifier to identify the three types of functional connectivity-based signatures: the state patterns (Figure 2), the trait patterns (Figure 3), and the compensatory patterns (Figure 4).

The identified state patterns related to the default mode network (DMN, mainly containing the parahippocampal gyrus, anterior cingulate cortex (ACC), hippocampus, thalamus, posterior cingulate cortex (PCC), medial prefrontal cortex, angular gyrus, rectus gyrus, precuneus and middle temporal gyrus), the dorsolateral prefrontal cortex-parietal executive control network (ECN) and the cerebellum. In the state patterns, several brain regions exhibited greater weights than others (i.e., the hippocampus, the ACC, the PCC, the medial prefrontal cortex, the middle temporal gyrus, the parietal gyrus and some cerebellar regions). For the trait patterns, functional connectivities between the cerebellum and the prefrontal lobe, the middle temporal gyrus, the thalamus and the middle temporal poles exhibited high discriminative power. In the compensatory patterns, connectivities related to the right precuneus, the left middle temporal gyrus, the left angular and the left rectus showed higher discriminative power.

## Discussion

### Multiclass pattern analysis

To the best of our knowledge, our study provided a novel use for a multiclass pattern analysis method based on rs-fMRI to investigate the functional connectivity patterns of the patients with schizophrenia, their healthy siblings and healthy controls.

Multivariate pattern analysis methods can not only find potential neuroimaging-based biomarkers to differentiate between patients and healthy controls at the individual subject level, but can also potentially detect exciting spatially distributed information to further highlight the neural mechanisms underlying the behavioral symptoms of psychiatry disorders. Previous studies have used multivariate pattern analysis to explore structural and functional alterations in schizophrenia and have obtained satisfactory correct classification rates [7,11,34,35]. Our previous study used multiclass pattern analysis to investigate the brains of patients with schizophrenia and their healthy siblings and suggest that healthy siblings may have a potentially higher risk for developing schizophrenia compared with the general population [4]; however, due to the limitation of nonlinear learning algorithms, this study failed to reveal potential neuroimaging-based biomarkers. In our current study, we used linear multiclass pattern analysis to address this issue and to focus on exploring the neuroimaging-based biomarkers. Our classification accuracy for these three groups is 62.0%, which is significantly above the chance level of 33.3%. Permutation tests indicate that the one-against-rest multiclass classifier learned the relationship between the data and the labels with a probability of error of <0.001. These results suggested that our methods can capture discriminative resting-state functional connectivity patterns among patients with schizophrenia, their healthy siblings and healthy controls. From Table 2, we found that the classification accuracy of healthy siblings of schizophrenic patients was comparatively lower and that these siblings were more likely than healthy controls to be misclassified as patients with schizophrenia, suggesting that the healthy siblings show a potentially higher risk for developing schizophrenia compared with the general population, which was consistent with previous findings [7,21]. In addition, the healthy siblings were much more likely to be misclassified as the healthy controls than as the patients with schizophrenia. This result might help to explain the normal daily behaviors exhibited in the healthy siblings of schizophrenic patients. Our findings also suggested that the connectivity patterns of the patients with schizophrenia, their healthy siblings and the healthy controls distributed differently throughout the entire brain rather than being restricted to a few specific brain regions.

### State connectivity pattern

The state patterns were mainly associated with the DMN, the ECN and the cerebellum. A great number of studies have demonstrated abnormalities in the DMN of patients with schizophrenia [2,36-38]. The DMN plays important roles in task-independent thought and self-referential processing, and altered connectivity within the DMN may have implications for cognition and task-related brain activity [39,40]. The hippocampus, the ACC, the temporal lobe, the PCC and the middle temporal gyrus have demonstrated alterations in connectivity with other brain regions of schizophrenic patients [20,40]. Ample evidence shows that the prefrontal cortex and the ACC play distinctive roles in cognitive functions, and the hippocampus has been suggested to be critical in memory formation [41]. Moreover, the ACC plays crucial roles in monitoring and detecting conflict in ongoing information processing [42], which may relate to cognitive functions. Dysfunctions of these above-mentioned brain regions might relate to the neuropathology of schizophrenia [43]. We also found discriminative connectivities between regions in the ECN and DMN areas, such as connectivities between the parietal gyrus and the ACC and PCC. Being somewhat consistent with our findings, impaired connectivities between components of the DMN and dorsolateral prefrontal cortex have been previously reported in schizophrenic patients [44]. The state patterns were also related to connectivities between the cerebellum and some cerebra regions. It has been suggested that the cerebellum is involved in cognitive and emotional activities [20]. Aberrant connectivities between the cerebellum and the cerebral cortex may be a part of the pathophysiology of schizophrenia [45].The state patterns indicated dysfunctional connectivities, which are associated with the manifestation of schizophrenia and might provide a clue about more complete pathophysiological mechanisms underlying this psychiatric disorder.

### Trait connectivity pattern

Trait patterns include connectivities across the DMN and other networks, as well as connectivities between the cerebellum and some cerebral regions. A few studies of schizophrenia have demonstrated impaired functional integration of the cerebellum [11]. Furthermore, a recent study reported impaired functional connectivities between the cerebellum and several cerebral regions, such as the thalamus, cingulate gyrus and inferior frontal gyrus, in patients with schizophrenia and their healthy siblings [46]. Though they are not totally consistent, we identified connectivities between the cerebellum and some cerebra regions, including the prefrontal lobe, the middle temporal gyrus and the thalamus. A previous study suggests that the prefrontal cortex dysfunction is associated with a higher risk for conversion to schizophrenia or expression of the illness [22]. We also discovered that patients with schizophrenia and their healthy siblings shared connectivities associated with the amygdala. These findings are compatible with a previous finding that revealed amygdala dysfunction in patients with schizophrenia and their relatives [37]. Although the healthy siblings were indistinguishable from healthy controls at the behavioral level, the trait patterns might help to explain the higher risk for healthy siblings than the general populations to develop schizophrenia.

### Compensatory connectivity pattern

We found that the healthy siblings exhibit unique connectivities within left regions of the DMN. For instance, connectivities have been associated with the left ACC. Previous sMRI studies demonstrated that white matter integrities in the left ACC are different in patients with schizophrenia and their healthy siblings [47]. An interesting observation is that connectivities related to the ACC were also identified in the state patterns. Because the ACC is critical for cognitive functions, an explanation for our findings could be that the ACC dysfunction may associate with the neuropathology of schizophrenia, whereas healthy siblings might benefit from the unique connectivities related to the left ACC compared with schizophrenic patients. Cautiously, we extended this explanation and came to the conclusion that the unique connectivities related to the left regions of the DMN might compensate for an increased genetic risk to develop schizophrenia for healthy siblings.

### Limitations

Due to the limited sample size and the relatively low classification accuracy, our findings need to be confirmed with a larger sample size in the future. Moreover, some of the patients with schizophrenia in this study were medicated. Previous studies suggest that antipsychotic treatments tend to change aberrant connectivity [41]. We are yet unable to exclude these possible effects of antipsychotic treatment.

## Conclusions

Despite these limitations, our classification results, to some extent, suggested that the multiclass pattern analysis methods can capture discriminative resting-state functional connectivity patterns among schizophrenic patients, their healthy siblings and healthy controls. We identified three types of functional connectivity-based signatures: i) relating to the state of having schizophrenia, ii) reflecting the genetic vulnerability to develop schizophrenia, and iii) underlying special brain connectivities by which healthy siblings overcome the genetic risk for developing schizophrenia. Our preliminary investigation suggested that resting-state functional connectivity is a promising feature for the classification among the schizophrenic patients, their healthy siblings and the healthy controls. In addition, on the basis of our experimental results, we saw some indication of differences in functional connectivity patterns in the healthy siblings of schizophrenic patients compared to other healthy individuals who have no relations with the patients. Our conclusions need to be considered with caution and confirmed by further investigations.

## Abbreviations

rs-fMRI: Resting-state functional magnetic resonance imaging; fMRI: Functional magnetic resonance imaging; sMRI: Structural magnetic resonance imaging; DTI: Diffusion tensor imaging; PCA: Principal component analysis; SVM: Support vector machine; LOOCV: Leave-one-out cross-validation; DMN: Default mode network; ECN: Executive control network; ACC: Anterior cingulate cortex; PCC: Posterior cingulate cortex.

## Competing interests

We declare that we have no competition and conflict of interests.

## Authors’ contributions

Author DH designed the study and wrote the protocol. Author HS managed the literature searches and analyses. Authors HZ and ZX recruited the participants and evaluated the symptom severity for the patients. Authors LLZ and YY undertook the statistical analysis, and author YY wrote the first draft of the manuscript. All authors contributed to and have approved the final manuscript.
